# Boosting the Biogenesis and Secretion of Mesenchymal Stem Cell-Derived Exosomes

**DOI:** 10.3390/cells9030660

**Published:** 2020-03-09

**Authors:** Jinli Wang, Emily E. Bonacquisti, Adam D. Brown, Juliane Nguyen

**Affiliations:** 1 Department of Biomedical Engineering, University at Buffalo, The State University of New York, Buffalo, NY 14214, USA; jinliwan@buffalo.edu; 2 Division of Pharmacoengineering and Molecular Pharmaceutics, Eshelman School of Pharmacy, University of North Carolina at Chapel Hill, Chapel Hill, NC 27599, USA; embona@live.unc.edu (E.E.B.); adamdb@email.unc.edu (A.D.B.)

**Keywords:** small molecules, exosome secretion, proteomics

## Abstract

A limitation of using exosomes to their fullest potential is their limited secretion from cells, a major bottleneck to efficient exosome production and application. This is especially true for mesenchymal stem cells (MSCs), which can self-renew but have a limited expansion capacity, undergoing senescence after only a few passages, with exosomes derived from senescent stem cells showing impaired regenerative capacity compared to young cells. Here, we examined the effects of small molecule modulators capable of enhancing exosome secretion from MSCs. The treatment of MSCs with a combination of N-methyldopamine and norepinephrine robustly increased exosome production by three-fold without altering the ability of the MSC exosomes to induce angiogenesis, polarize macrophages to an anti-inflammatory phenotype, or downregulate collagen expression. These small molecule modulators provide a promising means to increase exosome production by MSCs.

## 1. Introduction

Exosomes are nanosized (30–150 nm) extracellular vesicles released by almost all cell types and are critical in mediating cell–cell communication [1,2]. Exosomes are formed by the inward budding of the endosomal membrane, leading to the formation of multivesicular bodies (MVBs) [3]. The fusion of the MVB membrane with the plasma membrane releases the intraluminal vesicles (termed exosomes) into the extracellular space [4]. Alternatively, direct outward budding of the plasma membrane forms microvesicles (0.1–1 µm) [5]. Exosomes have emerged as promising carriers for cancer vaccines and drugs, as vehicles and mediators of cardiac regeneration, and as possible alternatives to cell-based therapies [6]. Mesenchymal stem cell (MSC)-derived exosomes have been shown to mediate tissue regeneration in a variety of diseases, including ischemic heart disease, lung injury, liver fibrosis, and cerebrovascular disease [7,8]. This is mainly due to their intrinsic regenerative capacity, in that they are able to induce angiogenesis, promote proliferation, prevent apoptosis, and inhibit inflammatory reactions [9]. In addition to their intrinsic biological functions, exosomes are also promising drug carriers due to their small size, excellent biocompatibility, and capacity to load specific and diverse therapeutic molecules including proteins, nucleic acids, and small molecules.

One limitation to using exosomes to their fullest potential is their limited secretion from cells, which constitutes a major bottleneck to efficient, large-scale exosome production. This is especially true for MSCs which, while able to self-renew, have limited expansion capability [10]. MSCs undergo senescence after only a few passages, and exosomes derived from senescent stem cells have impaired regenerative capacity compared to young cells [11]. MSCs have been transfected with lentiviruses carrying MYC to generate immortalized MSCs [10], but MYC is a proto-oncogene and thus could cause undesirable side effects if used therapeutically.

Therefore, enhancing exosome production is critical for both allogeneic and autologous exosome-based therapies because: (i) time is a limiting factor, since exosome-based therapies need to be administered as quickly as possible, particularly for cancer vaccination or to reduce the immunogenic effects caused by non-matching allogeneic cells; (ii) large quantities of exosomes are required for exosome-based therapies in a very short timeframe, and while upscaling of cell cultures is an option, this approach is limited by the growth kinetics and number of cells isolated from patients; and (iii) expanded exosome synthesis would allow the production of off-the-shelf therapeutics.

Different strategies have been reported to increase the production of exosomes, such as inducing hypoxia (1.3-fold enrichment in MSCs), overexpressing tetraspanin CD9 (2.4-fold enrichment in HEK293), or overexpressing hypoxia-inducible factor-1α (2.2-fold enrichment in MSCs) [12,13,14]. Other upscaling approaches has involved increasing the volume of cell culture from flasks to containers, bioreactors, or hollow fibers [15,16]. The objectives of this study were to: (i) identify a set of small molecules capable of increasing exosome production in MSCs, and (ii) determine if the small molecule modulators affected the exosomal composition and the intrinsic regenerative capacity of MSC exosomes. 

## 2. Materials and Methods 

### 2.1. Materials

Fenoterol hydrobromide and L-(−)-Norepinephrine-(+)-bitartrate were purchased from Sigma-Aldrich (St. Louis, MO, USA) (catalog No. F1016 and 489350). Forskolin was purchased from Cayman Chemical (Ann Arbor, MI, USA) (catalog No. 11018). N-Methyldopamine hydrochloride was purchased from Alfa Aesar (Tewksbury, MA, USA) (catalog No. J60306). Mephenesin was purchased from Ambeed (Chicago, IL, USA) (catalog No. A307218).

### 2.2. Extracellular Vesicle Isolation

Exosomes were isolated from passage P1-P6 human bone marrow-derived MSCs obtained from the American Type Culture Collection (ATCC PCS-500-012) and were cultured in MesenPRO RS™ Medium (GibcoTM, Gaithersburg, MD, USA). A total of 48 hours after compound treatment in exosome-depleted medium, exosomes were harvested using differential ultracentrifugation. The cell culture medium was centrifuged at 2000 rpm for 10 min to remove cells and debris. Then, the supernatants were centrifuged at 10,000× *g* for 30 min at 4 °C and the supernatants were transferred to ultracentrifuge tubes (Beckman Coulter, Brea, CA, USA) and diluted with phosphate-buffered saline (PBS). Tubes were centrifuged twice at 100,000× *g* for 70 min at 4 °C with a Beckman ultracentrifuge (32Ti rotor from Beckman Coulter, Indianapolis, IN, USA). Supernatants were removed and the pellets resuspended in phosphate-buffered saline (PBS) between centrifugations. Finally, the pellets were suspended in PBS for downstream analysis.

### 2.3. Exosome Characterization by Nanoparticle Tracking Analysis (NTA) 

The size distribution and concentration of exosomes were characterized by nanoparticle tracking analysis (NTA) as previously described [17]. Briefly, exosomes were diluted in PBS and measured three times in three different areas for each sample, with the average used to determine exosome concentration.

### 2.4. Exosome Characterization by Transmission Electron Microscopy (TEM)

To prepare samples for electron microscopy, formvar/carbon-coated grids, were glow-discharged for 15 s. The samples were adsorbed to grids by floating the grids for 30 s on droplets containing exosomes diluted in PBS. Excess adherent sample was removed from the grid by wicking with filter paper. A negative stain was applied in the form of 2% uranyl acetate for 30 s. Excess adherent negative stain was removed from grid by wicking with filter paper. The samples were imaged using a FEI Tecnai T12 Transmission Electron Microscope (FEI Company, Hillsboro, OR, USA) and a Gatan Rio 16 CMOS Camera (Gatan, Inc., Pleasanton, CA, USA). Exosome size distributions were determined by manually measuring the major axis of the ellipse that best approximates the shape of 101–127 exosomes per sample group using ImageJ.

### 2.5. Western Blotting

Exosome pellets were re-suspended and lysed with RIPA buffer (Santa Cruz Biotechnology, Dallas, TX, USA), incubated at 4 °C for an additional 15 min for complete lysis and were combined with 4× LDS buffer (Bio-Rad Laboratories, Hercules, CA, USA). Samples were heated to 95 °C for 5 min and then analyzed on a 4–12% gel (Bio-Rad Laboratories, Hercules, CA, USA) using SDS running buffer. Transfer onto PVDF membrane was performed at 100 V for 120 min. The blots were incubated with anti-human CD63^+^ primary antibody (1:1000, Cat# 556019; Becton Dickinson, Franklin Lakes, NJ, USA) or CD9 primary antibody (1:1000, Cat# 555370; Beckton Dickinson) overnight at 4 °C and visualized using the Bio-Rad ChemiDoc MP Imaging System (Bio-Rad Laboratories, Hercules, CA, USA).

### 2.6. MTT Assay

Cellular viability was analyzed using the 3-(4,5-dimethylthiazol-2-yl)-2,5-diphenyl tetrazolium bromide (MTT) assay as previously described [18,19,20]. MSCs were seeded into 96-well plates and cultured overnight. Cells were treated with different concentrations of either compounds or exosomes for 24 h. MTT solution was added to the cells and incubated at 37 °C for 3.5 h. Acidified isopropanol was added to dissolve the purple crystals, the plate was shaken for 15 min at RT, and the absorbance was read at 590 nm using an Epoch microplate spectrophotometer (BioTek, Winooski, VT, USA). The background was removed by subtracting the absorbance recorded from a well containing no cells. The viability was calculated by dividing the absorbance from non-treated wells, which was set to 1.

### 2.7. Collagen Expression Assay

Primary human cardiac fibroblasts were obtained from ScienCell (Cat# 6330, ScienCell Research Laboratories Inc., Carlsbad, CA, USA). Passage number P3-P5 cells were taken, plated into 24-well plates, cultured in fibroblast medium 2 (Cat# 2331, ScienCell, Carlsbad, CA, USA) containing 5% fetal bovine serum (FBS), and were grown to 80% confluency. The cells were treated with 8 µg/mL exosomes. After 12 h, the cells were stimulated with an additional 500 μL of complete medium containing TGF-β (Peprotech, Rocky Hill, NJ, USA) at a final concentration of 10 ng/mL. To assess collagen production, the cells were lysed for RNA extraction after 48 h according to the TRIzol protocol (Thermo Fisher Scientific, Waltham, MA, USA). cDNA was synthesized with the First Strand cDNA Synthesis Kit (New England BioLabs, Ipswich, MA, USA) and was analyzed for the expression of collagen I (COL1A1) (primer sequences: forward GGGCAAGACAGTGATTGAATA and reverse ACGTCGAAGCCGAATTCCT), and GAPDH (forward CAAGGTCATCCATGACAACTTTG and reverse GTCCACCACCCTGTTGCTGTAG) by quantitative reverse transcription polymerase chain reaction (RT-qPCR) using the SYBR Supermix (Bio-Rad Laboratories) on a CFX Connect Real-Time PCR Detection System (Bio-Rad Laboratories).

### 2.8. Angiogenesis Assay

Primary human umbilical vein endothelial cells (HUVECs) were obtained from Gibco (Cat#: C0035C) and cultured in Medium 200 (Cat#: M200500, Thermo Fisher Scientific, Waltham, MA, USA) containing large vessel endothelial supplement (Cat#: A1460801, Thermo Fisher Scientific). HUVECs were incubated with 7.5 μg/mL of MSC-derived exosomes or compounds for 24 h. Then, HUVECs were plated into wells coated with Geltrex (Cat#: A1413201, Thermo Fisher Scientific) and cultured overnight. Seventeen hours after plating, HUVECs were stained with calcein and imaged at em/ex 488/505 with a MiniMax Imager (Molecular Devices, Sunnyvale, CA, USA). Angiogenesis was analyzed with ImageJ software using the angiogenesis analyzer (Bethesda, MD, USA).

### 2.9. Bone Marrow-Derived Macrophage (BMDM) Isolation and Culture

Bone marrow was harvested from the femurs and tibias of C57BL/6 mice as previously described [21]. After isolation and centrifugation, bone marrow cells were resuspended in DMEM/F12-10, frozen in DMEM/F12-40 with 10% dimethyl sulfoxide (DMSO) and were stored in liquid nitrogen until further use. The cells were thawed, washed once, and resuspended in macrophage complete medium (DMEM/F12, 10% FBS, 1% penicillin/streptomycin, 100 U/mL recombinant murine M-CSF; Peprotech, Rocky Kill, NJ, USA; Cat# 315-02). A total of 5 mL of macrophage complete medium was added on day 3. On day 7, the cells were used for polarization assays.

### 2.10. Macrophage Polarization Assay 

BMDMs were treated with 16 µg/mL exosomes or different doses of compounds. Twenty-four hours after treatment, pro- and anti-inflammatory markers were quantified by RT-PCR. Briefly, cells were lysed with TRIzol reagent to extract RNA. cDNA was synthesized with the First Strand cDNA Synthesis Kit, as above, and was analyzed for the expression of *iNOS* (forward CACCTTGGAGTTCACCCAGT, reverse ACCACTCGTACTTGGGATGC), *IL6* (forward ACTTCACAAGTCGGAGGCTT, reverse TGGTCTTGGTCCTTAGCCAC), *Arg1* (forward GTGAAGAACCCACGGTCTGT, reverse CTGGTTGTCAGGGGAGTGTT), *CD206* (forward CAAGGAAGGTTGGCATTTGT, reverse CCAGGCATTGAAAGTGGAGT), and beta-actin (Actb) (forward GCCTTCCTTCTTGGGTATGG, reverse CAGCTCAGTAACAGTCCGCC) by RT-qPCR using SYBR Supermix (Bio-Rad Laboratories) on a CFX Real-Time PCR Detection System (Bio-Rad Laboratories). 

### 2.11. Mechanism Study

After 48 h of compound treatment, MSCs were lysed and RNA were extracted with Trizol according to the manufacturer’s protocol (Invitrogen, Carlsbad, CA, USA). cDNAs were synthesized using the ProtoScript^®^ II First Strand cDNA Synthesis Kit (New England BioLabs, Ipswich, MA, USA) and were analyzed for the expression of the exosome production-related genes listed in Supplementary Table S1 by RT-PCR on a CFX Connect Real-Time System (Bio-Rad Laboratories). 

### 2.12. Mass Spectrometric Analysis of Exosomes

MSCs were treated with norepinephrine and N-methyldopamine (NE + MeDA) for 24 h prior to switching to serum-free media for 24 h prior to exosome isolation. Exosomes were isolated using differential ultracentrifugation as described previously. Exosome samples were brought up to 30 µL in LDS Sample Loading Buffer (Invitrogen, Waltham, MA, USA), and then run into a 4–12% SDS-Page gel for a short period of time to remove contaminants. After staining with SimplyBlue SafeStain (Invitrogen), these regions were excised, cut into 1mm cubes, de-stained, then reduced and alkylated with dithiothreitol (DTT) and iodoacetamide (IAA), respectively (Sigma, St. Louis, MO, USA). Gel pieces were dehydrated with acetonitrile and digested with 10 ng/µL Trypsin in 50 mM ammonium bicarbonate for thirty min at room temperature, then placed at 37 °C overnight. Exosomal peptides were extracted the next day by the addition of 50% acetonitrile and 0.1% trifluoroacetic acid (TFA), and were then dried down in a CentriVap concentrator (Labconco, Kansas City, MO, USA). Peptides were desalted with homemade C18 spin columns, dried again, and reconstituted in 0.1% TFA.

Peptides were injected onto a 30-cm C18 column with 1.8 µm beads (Sepax, Newark, NJ, USA), with an Easy nanoLC-1200 HPLC (Thermo Fisher, Waltham, MA, USA), connected to an Orbitrap Fusion Lumos mass spectrometer (Thermo Fisher). Solvent A was 0.1% formic acid in water, while solvent B was 0.1% formic acid in 80% acetonitrile. Ions were introduced to the mass spectrometer using a Nanospray Flex source operating at 2 kV (Thermo Fisher, Waltham, MA, USA). Peptides were eluted off the column using a multi-step gradient that began at 3% B and held for 2 min, quickly ramped to 10% B over 6 min, increased to 38% B over 95 min, then ramped to 90% B in 5 min and was held there for an additional 3 min to wash the column. The gradient then returned to starting conditions in 2 min and the column was re-equilibrated for 7 min, for a total run time of 120 min. The flow rate was 300 nL/min throughout the run. The Fusion Lumos was operated in data-dependent mode with a cycle time of 2 s. The full scan was done over a range of 375–1400 *m*/*z*, with a resolution of 120,000 at *m*/*z* of 200, an automatic gain control (AGC) target of 4e5, and a maximum injection time of 50 ms. Peptides with a charge state between 2–5 were selected for fragmentation. Precursor ions were fragmented by collision-induced dissociation (CID) using a collision energy of 30 and an isolation width of 1.1 *m*/*z*. MS2 scans were collected in the ion trap with the scan rate set to rapid, a maximum injection time of 35 ms, and an AGC setting of 1e4. Dynamic exclusion was set to 45 s to allow the mass spectrometer to fragment lower abundant peptides.

### 2.13. Bioinformatic Data Analysis

Raw data was searched with the SEQUEST search engine within the Proteome Discoverer software platform, version 2.2 (Thermo Fisher), using the UniProt human database. Trypsin was selected as the enzyme allowing up to 2 missed cleavages, with an MS1 mass tolerance of 10 ppm, and an MS2 mass tolerance of 0.6 Da. Carbamidomethyl on cysteine was selected as a fixed modification. Oxidation of methionine was set as a variable modification. Percolator was used as the FDR calculator, filtering out peptides with a q-value greater than 0.01. Label-free quantitation was performed using the Minora Feature Detector node (Thermo Fisher Scientific, Waltham, MA, USA), with a minimum trace length of 5. The Precursor Ions Quantifier node (Thermo Fisher Scientific, Waltham, MA, USA) was then used to calculate protein abundance ratios, using only unique and razor peptides. The pairwise based method was employed to calculate the protein ratios, which uses a protein’s median peptide ratio to determine the protein ratio. Normalization was done using the Total Peptide Amount method. A comparison of the treated and non-treated groups was analyzed using Graphpad Prism 8 (GraphPad, San Diego, CA, USA). Protein pathway analyses were conducted using a combination of Kyoto encyclopedia of genes and genomes (KEGG), Reactome, and Panther gene hits. Protein interactors were determined utilizing StringDB.org [22] and uploaded into Cytoscape 3.2.1 [23], before being fed into ClueGo 2.5.4 for visualization [24]. Proteins that did not fit these parameters were removed from subsequent analyses due to lack of interaction evidence. Only gene hits with a statistical significance of *p* < 0.05 with a Bonferroni step-down correction were considered for further discussion.

## 3. Results

### 3.1. Effects of Small Molecules on Exosomal Production Efficiency

**Figure 1:**
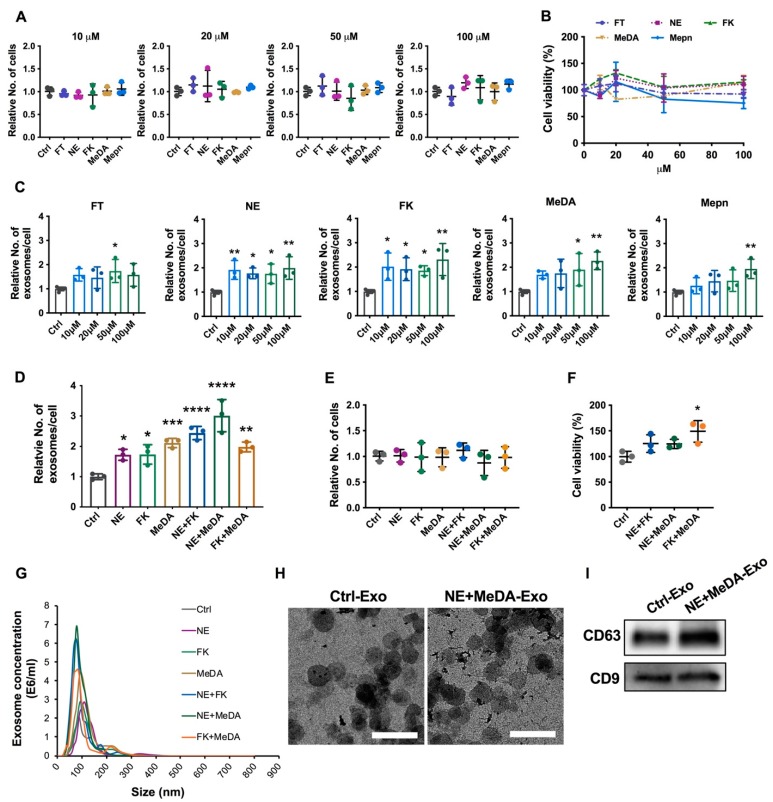
Effects of small molecules on MSC cell viability and exosome production efficiency. (**A**) Cell proliferation rate. (**B**) The cell viability and metabolism of mesenchymal stem cells (MSCs) treated with different doses of compounds measured by the 3-(4,5-dimethylthiazol-2-yl)-2,5-diphenyl tetrazolium bromide (MTT) assay. (**C**) The production efficiency of exosomes derived from MSCs treated with different doses of compounds. (**D**) The production efficiency of exosomes derived from different MSCs treated with combinations of compounds. (**E**) The cell proliferation rate of MSCs in response to treatment with small molecule modulators. (**F**) The cell viability and metabolism of MSCs after treatment with small molecule modulators measured by the MTT assay. (**G**) The size distribution of exosomes by nanoparticle tracking analysis (NTA) in response to the treatment of MSCs with small molecule modulators. (**H**) Transmission electron microscopy (TEM) images of exosomes, scale bar 200 = nm, Exo: Exosomes. (**I**) CD63 and CD9 exosomal surface marker protein expression by Western Blotting. One-way ANOVA with Dunnett’s multiple comparisons test; * *p* < 0.05, ** *p* < 0.01, *** *p* < 0.001, **** *p* < 0.0001, *n* = 3–6 each group. FT: fenoterol; NE: norepinephrine; FK: forskolin; MeDA: N-methyldopamine; Mepn: mephenesin.

Five compounds were selected to assess if they could stimulate MSCs to enhance their exosome production: four FDA-approved drugs (fenoterol, norepinephrine, N-methyldopamine, and mephenesin) and the other nutritional supplement (forskolin). The small molecule modulators were selected from a recent screening in prostate cancer cells [25]. To determine a range of non-toxic concentrations, MSCs were treated with the five different compounds at concentrations between 10 and 100 µM. Two independent assays were used to determine the effects of the compounds on cells: (a) cell and metabolic activity as determined by the MTT assay, and (b) total cell number. The treatment of MSCs with these compounds at the tested range of concentrations did not affect MSC proliferation and had no obvious cytotoxicity (Figure 1A,B).

After confirming that all five compounds were well tolerated by MSCs even at high concentrations, the effects of these small molecules on MSC exosome production efficiency were examined. All the tested doses of norepinephrine (NE) and forskolin (FK) increased exosome production significantly and by similar levels (1.75- to 2-fold for norepinephrine and 1.9- to 2.3-fold for forskolin, *p* < 0.05). At a concentration of 50 µM, fenoterol (FT) increased exosome production by approximately 1.7-fold, *p* < 0.05). N-Methyldopamine (MeDA) and mephenesin (Mepn) increased exosome production in a dose-dependent manner, with 100 µM showing the greatest effect on exosome production (~2.3-fold for MeDA, *p* < 0.001; and ~2-fold for Mepn, *p* < 0.01) (Figure 1C). 

Combining norepinephrine with forskolin and norepinephrine with N-methyldopamine further enhanced exosome production by ~2.5-fold and ~3-fold, respectively (Figure 1D). Adding forskolin in combination with N-methyldopamine did not further increase exosome production (Figure 1D). While the combination treatment did not affect overall cell counts (Figure 1E), they increased the metabolic activity of MSCs by ~1.5-fold, as measured by the MTT assay (Figure 1F).

Exosomes derived from non-treated MSCs and MSCs treated with the small molecule compounds measured 80 to 100 nm in size (Figure 1G). By NTA, the exosomes derived from MSCs treated with a combination of NE and MeDA were 103 ± 8 nm in size, while exosomes derived from untreated MSCs were slightly smaller at 92 ± 14 nm in size (Figure 1G). Similar results were obtained by TEM: exosomes isolated from MSCs treated with a combination of NE and MeDA were 100 ± 28 nm in size and exosomes derived from untreated MSCs were 87 ± 12 nm in size (Figure 1H). A Western Blot was used to confirm that the isolated exosomes expressed the exosome-specific tetraspanins CD63 and CD9 (Figure 1I). The exosome concentration was determined by both NTA and Bradford protein assay in this study. The equivalent amounts were used as follows: 1 µg exosome is equivalent to 4.4 ± 0.2 × 10^8^ exosomes.

### 3.2. Downregulation of Collagen Expression

Exosomes derived from MSCs have been shown previously to have intrinsic anti-fibrotic effects [26]. To determine if compound treatment affected the intrinsic biological effects of the produced MSC exosomes, cardiac fibroblasts were incubated with exosomes derived from untreated and treated MSCs.

**Figure 2:**
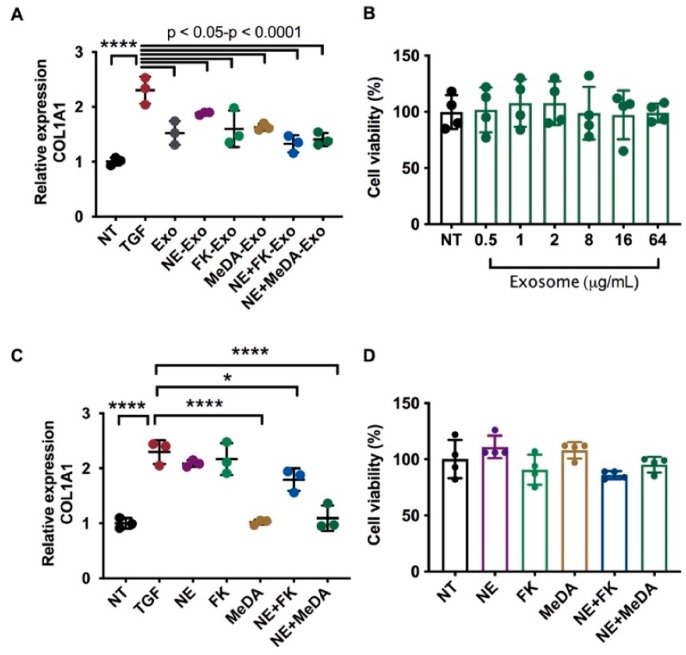
Effects of exosomes derived from MSCs treated with small molecule modulators on collagen expression. (**A**) COL1A1 expression in human cardiac fibroblasts (HCF) incubated with exosomes isolated from MSCs treated with small molecule modulators as determined by quantitative reverse transcription polymerase chain reaction (RT-qPCR). (**B**) MTT assay of MSC-derived exosomes on HCFs. (**C**) *COL1A1* expression in human cardiac fibroblasts (HCF) incubated with small molecule modulators. (**D**) MTT assay of compound treatment on HCFs. One-way ANOVA with Dunnett’s multiple comparisons test, * *p* < 0.05, ** *p* < 0.01, *** *p* < 0.001, **** *p* < 0.0001; *n* = 3–4.

To induce collagen expression, cardiac fibroblasts were stimulated with TGF-β (Figure 2A). Untreated MSC-derived exosomes significantly decreased TGF-β-induced collagen expression from 2.3-fold to ~1.5-fold (*p* < 0.001). All the compound-treated MSC-derived exosomes decreased TGF-β-induced collagen expression, indicating that compound-treated MSC-derived exosomes preserve their intrinsic effects (Figure 2A). The MTT assay showed that MSC-derived exosomes did not affect fibroblast cell viability, indicating that the collagen-suppressive effects were not due to cytotoxicity (Figure 2B). We further tested the direct effect of the compounds on collagen expression and found that MeDA and combinatorial compound treatment significantly inhibited TGF-β-induced collagen expression but did not affect cell viability (Figure 2C,D).

### 3.3. Macrophage Polarization 

**Figure 3:**
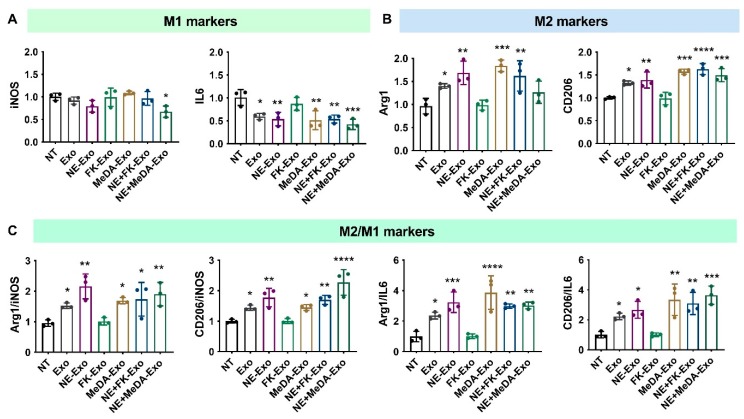
Polarization of macrophages with compound-treated MSC-derived exosomes. (**A**) Inflammatory macrophage (M1) markers *iNOS* and *IL6*. (**B**) Anti-inflammatory macrophage (M2) markers *Arg1* and *CD206*. (**C**) M2/M1 ratios. One-way ANOVA with Dunnett’s multiple comparisons test, * *p* < 0.05, ** *p* < 0.01, *** *p* < 0.001, **** *p* < 0.0001; *n* = 3.

In addition to their ability to inhibit collagen expression, MSC-derived exosomes polarize macrophages to the anti-inflammatory M2 phenotype. As shown in Figure 3, MSC-derived exosomes had negligible effects on the M1 marker *iNOS* but significantly decreased *IL6* expression compared to untreated cells (NT group). Similar to exosomes derived from untreated MSCs, exosomes derived from MSCs treated with the small molecules did not alter *iNOS* expression but significantly decreased *IL6* levels (Figure 3A). Except for the forskolin-treated group, exosomes derived from non-treated MSCs and MSCs treated with norepinephrine and N-methyldopamine increased anti-inflammatory M2 markers *Arg1* and *CD206* (Figure 3B). Almost all the MSC-derived exosomes increased the M2/M1 ratio, but those induced by forskolin failed to significantly increase *Arg1*/*iNOS*, *Arg1*/*IL6*, and *CD206*/*IL6* (Figure 3C). These results suggest that the treatment of MSCs with the compounds did not alter the intrinsic effects of exosomes on macrophage polarization. 

We further tested the direct effect of compounds alone at the full doses that were used to treat MSC, and at 10-fold and 100-fold dilutions to determine dose effects [27]. The results show that compound treatment did not alter *iNOS* expression level, while high doses of NE and MeDA significantly decreased *IL6* expression compared to untreated cells. High doses of NE and MeDA significantly increased M2 markers (*Arg1* and *CD206*). FK did not change the expression of macrophage markers tested (Supplementary Figure S3). 

### 3.4. Angiogenesis Effect

**Figure 4:**
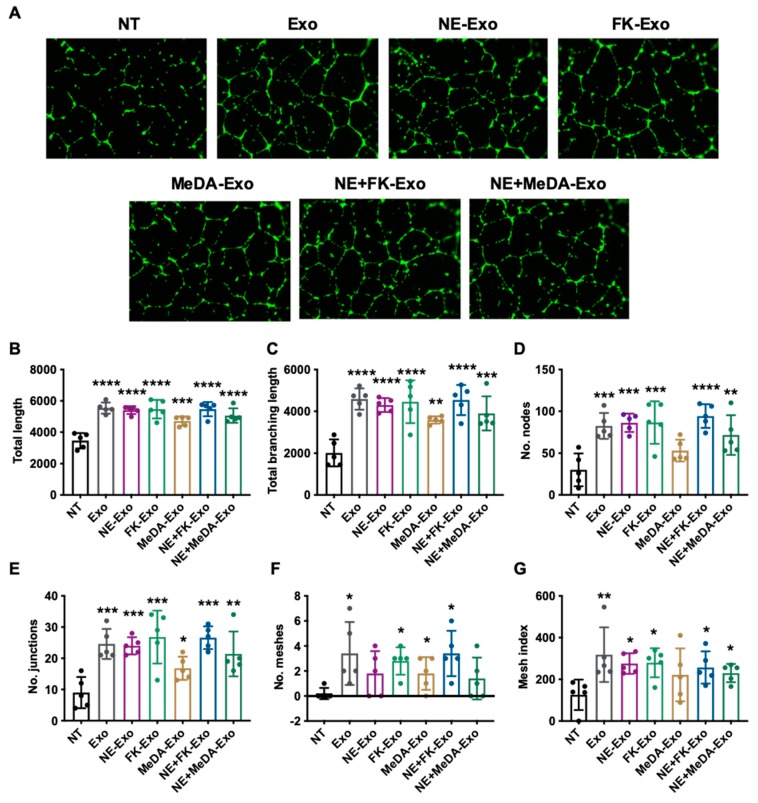
Tube formation assay. (**A**) Representative images of the angiogenesis effect of compound-treated MSC-derived exosomes. (**B**–**G**) Quantification of angiogenesis using ImageJ. (**B**) Total length. (**C**) Total branching length. (**D**) The number of nodes. (**E**) The number of junctions. (**F**) The number of meshes. (**G**) The mesh index. One-way ANOVA with Dunnett’s multiple comparisons test, * *p* < 0.05, ** *p* < 0.01, *** *p* < 0.001, **** *p* < 0.0001; *n* = 5.

To assess if compound-induced exosomes had altered ability to induce angiogenesis, HUVECs were incubated with exosomes derived from untreated MSCs and MSCs treated with the small molecules. Similar to HUVECs treated with exosomes derived from untreated MSCs, exosomes derived from treated MSCs significantly increased parameters for tube formation, such as increased total length, total branching length, the number of junctions, the number of nodes, the number of meshes, and the mesh index (Figure 4). Exosomes induced by small molecules preserved their intrinsic pro-angiogenic function. 

### 3.5. Effects of Small Molecule Modulators on Genes Affecting Exosome Biogenesis and Secretion

Cellular exosome production involves several processes that include the inward budding of the late endosomal membrane to form intraluminal vesicles within multivesicular bodies (MVBs). This is followed by the fusion of MVBs with the plasma membrane prior to exosomal release into the extracellular milieu [28].

**Figure 5:**
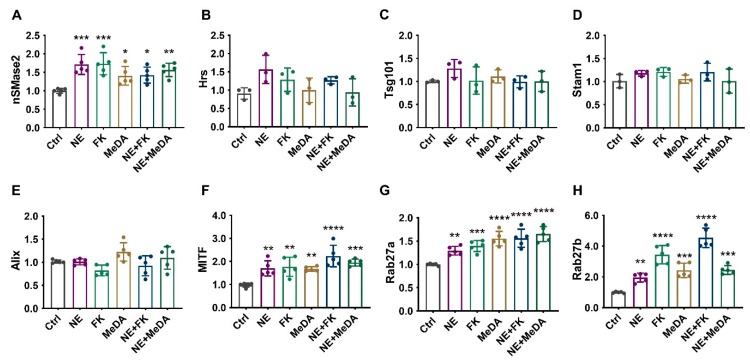
The effect of small molecule compounds on exosomal production-related pathways after 48 h treatment. RT-PCR quantification of (**A**) nSMase2 (*SMPD3*), (**B**) *Hrs*, (**C**) *Tsg101*, (**D**) *Stam1*, (**E**) *Alix*, (**F**) microphthalmia-associated transcription factor (*MITF*), (**G**) *Rab27a*, and (**H**) *Rab27b*. One-way ANOVA with Dunnett’s multiple comparisons test, * *p* < 0.05, ** *p* < 0.01, *** *p* < 0.001, **** *p* < 0.0001; *n* = 3–5.

We first assessed the effects of the small molecule antagonists on neutral sphingomyelinase (nSMase2; *SMPD3*) expression, because it has been shown to be a key lipid metabolic enzyme in early exosome formation [28]. Ceramide is one of the main regulators of exosome formation and it is formed after the hydrolytic removal of the phosphocholine moiety of sphingomyelin by sphingomyelinase. Ceramide triggers the inward budding and formation of intraluminal vesicles resulting in multivesicular bodies [29]. The treatment of MSCs with small molecule modulators significantly upregulated nSMase2 by 1.5- to 2-fold (Figure 5A). In contrast, the small molecule modulators did not affect the expression of *Hrs*, *Tsg101*, *Stam1*, or *Alix*, which are known to regulate the endosomal sorting complex required for transport (ESCRT)-dependent biogenesis of exosomes (Figure 5B–E) [30].

Next, we assessed the effects of the small molecule modulators on microphthalmia-associated transcription factor (*MITF*). *MITF* has been reported to play an important role in exosome generation and is known to increase the expression of late endosomal proteins such as Rab7 and Rab27a, which are key proteins in exosomal biogenesis and secretion [31,32]. The small molecule modulators significantly upregulated *MITF* by 1.5- to 2-fold (Figure 5F).

Finally, we assessed how the small molecule modulators affected *Rab27a* and *Rab27b*. Rab family proteins have been reported to promote exosomal transportation and secretion. Rab27a/b have also been reported to be required for (a) MVB distribution to the cell periphery, and (b) MVB docking at the plasma membrane for exosome exocytosis [33]. Upon stimulation with small molecule modulators, *Rab27a* and *Rab27b* were increased ~1.3 to 5-fold (Figure 5G,H).

### 3.6. Effects of Small Molecule Modulators on Exosomal Protein Composition

**Figure 6:**
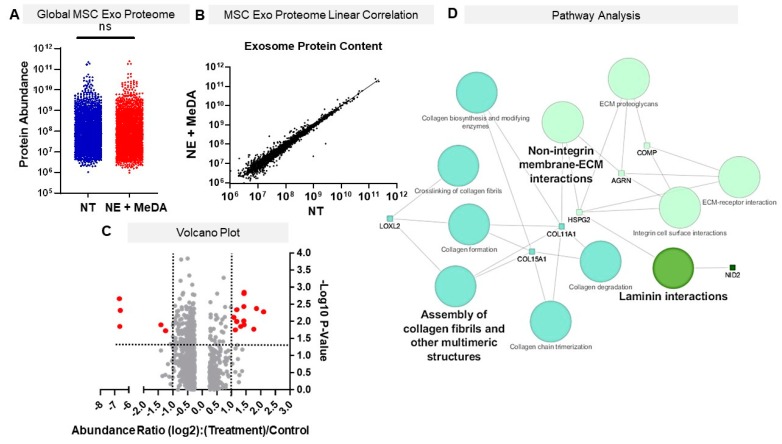
Proteomic profile of MSC exosomes 48h after norepinephrine and N-methyldopamine (NE + MeDA) treatment. (**A**) Raw abundance values from the NT and NE + MeDa groups showed no statistical difference using a paired t-test across samples. (**B**) Linear regression analysis comparing non-treated MSCs (NT) and MSCs treated with NE + MeDA. (**C**) Data visualized using a volcano plot with red dots indicating statistically significantly different proteins with an abundance change of 2-fold or higher. (**D**) Cytoscape 3.2.1 visualization of Kegg, Reactome, and Panther Pathway hits of differentially expressed proteins using ClueGo v.2.5.4. The font size is mapped to the *p* value with Bonferroni step down correction, and the node size is mapped to number of statistically significant gene hits.

Because small molecule treatment might not only modulate exosome secretion but could also affect exosome composition, proteomic analysis was performed on exosomes derived from untreated MSCs and MSCs treated with a combination of norepinephrine and N-methyldopamine, which had the greatest effect on exosome secretion. In order to detect differences in protein abundance, proteomic data were analyzed using global and individual statistical comparisons. There were no statistically significant differences in total protein composition and the amounts between exosomes derived from unmodified MSCs and exosomes derived from MSCs treated with norepinephrine and N-methyldopamine (Figure 6A,B). Of the 2159 proteins identified in each sample, seven (0.3%) were statistically significantly downregulated. A total of 14 (0.6%) of the total protein content was statistically significantly higher in abundance (Figure 6C, Supplementary Figure S2). Each of these proteins was enriched between ~2 and ~5 fold over the untreated. 

From the control of these 14, only seven (LOXL2, COL15A1, COL11A1, HSPG2, AQRN, NID2 and COMP) were upregulated above the cumulative average protein abundance (Supplementary Figure S2). None of the downregulated proteins were at or above the cumulative average, so this, along with the lack of statistical significance, excluded them from pathway analysis (Supplementary Figure S2). The combinatorial pathway and interaction analysis were stringently annotated and analyzed with the pathway visualization software ClueGo. Three pathway databases, Panther, KEGG, and Reactome, were used for analyses in order to create a comprehensive pathway visualization. Statistically significant pathways are visualized in Figure 6D, with the font and node size corresponding to statistical significance and the number of pathway gene hits, respectively. The pathways that were most represented were the assembly of collagen fibrils and other multimeric structures, laminin interactions, and non-integrin membrane-extracellular matrix (ECM) interactions. 

## 4. Discussion

Exosomes derived from MSCs are known to have intrinsic tissue regenerative effects; that is, they induce angiogenesis and cellular proliferation and inhibit fibrosis [9]. However, their widespread therapeutic use as drug carriers is currently hampered by their limited cellular secretion [15]. Exosome yield using a routine cell culture is generally low and could significantly slow production, which is of particular concern if exosomes need to be derived rapidly from the patient’s own cells for personalized therapy. Having the means to significantly increase exosome secretion will be a highly important tool for the development of exosomes as drug carriers and their related clinical applications.

To date, the upscaling of exosome production has involved increasing the volume of cell culture from flasks to containers, bioreactors, or hollow fibers [15,16]. These methods require substantial volumes and are impractical for large-scale production. Thus, alternative complementary or stand-alone methods to existing approaches would be highly useful.

GW4869 and manumycin-A were the first and few molecules known to inhibit exosome secretion [34,35]. Recently, prostate cancer cells were screened with small molecules to test their effects on exosome production [25,35], but the molecules identified in that screen (tipifarnib, nitrefazole, pentetrazol, etc.) have not been tested or shown to work in other cell types [25], despite evidence of cell type-specific effects of small molecules on exosome secretion [35]. Here, we assessed the effects of a set of small molecules on exosome production from MSCs.

Five compounds (fenoterol, norepinephrine, forskolin, N-methyldopamine, and mephenesin) were selected from a screen previously reported to enhance exosome secretion in C4-2B castration-resistant prostate cancer cells [25]. While these compounds induced a 3.5- to 5.7-fold increase in exosome secretion in C4-2B prostate cancer cells at 10 µM concentrations, fenoterol, N-methyldopamine, and mephenesin failed to enhance exosome secretion at low doses in MSCs and only slightly enhanced exosome secretion at significantly higher doses (50 µM for fenoterol, 100 µM for mephenesin, and 50 µM for N-methyldopamine). Norepinephrine and forskolin were reported to increase exosome secretion in C4-2B cells by 4.6- and 5.7-fold. When tested in MSCs, exosome secretion was enhanced by only two-fold. When dual combinations of the small molecules were tested, norepinephrine co-dosed with N-methyldopamine had the greatest effects on exosome secretion in MSCs with a three-fold enhancement. This strongly suggests that exosome secretion is a cell-specific process and that small molecule modulators do not necessarily have the same effects on all cell types [25,35].

We showed that enhanced exosome secretion is not due to an increase in cell number after small molecule modulator treatment but could be the result of enhanced metabolic activity [36]. This is consistent with our target prediction analysis that revealed that forskolin mainly binds to proteins related to energy (ATP and glucose) production (Supplementary Figure S1). N-Methyldopamine binds to the dopamine and adrenergic receptors, and norepinephrine binds to adrenergic receptors, whose activation has been shown to increase cellular metabolism (Supplementary Figure S1) [37].

We chose three representative assays to assess if the small molecule modulators affected the intrinsic regenerative effects of MSC exosomes [18]. Exosomes derived from MSCs treated with small molecule modulators showed a similar inhibition of collagen expression levels in cardiac fibroblasts as the exosomes derived from unmodified MSCs. Similarly, there were no differences in their ability to polarize macrophages to the M2 anti-inflammatory phenotype. Finally, in a tube formation assay, MSC exosomes derived from MSCs treated with small molecule modulators showed comparable angiogenic ability as control exosomes. This strongly indicates that chemically induced exosomes preserve their biological functions. In line with this, small molecule treatment of MSCs did not dramatically affect exosomal protein composition, in agreement with findings where an increase in Rab27a and Rab27b in HeLa cells did not affect exosomal protein composition [33]. The increased metabolic activity of the MSCs after small molecule treatment remained independent of exosome protein biogenesis, further showing the utility of the treatment as a powerful biochemical tool for increasing the throughput of exosome research. 

We further tested the direct effects of the small molecule modulators on collagen expression, macrophage polarization and angiogenesis. First, we found that MeDA treatment significantly inhibited TGF-β-induced collagen expression but not cell viability. (Figure 2C,D). Second, NE and MeDA significantly increased M2-macrophage markers (*Arg1* and *CD206*) and decreased *IL6*. (Supplementary Figure S3). Third, NE and FK treatment did not affect tube formation, while 100 μM MeDA alone or co-dosed with NE significantly impaired angiogenesis (Supplementary Figure S3). Given that the exosomes derived from MSCs treated with NE and MeDA had similar biological effects compared to exosomes derived from non-treated cells, it is highly likely that NE and MeDa were incorporated at very low amounts into the exosomes. This is in line with studies showing that the pre-treatment of cells with small molecules results in extremely low exosomal loading [27]. For example, Pascucci et al. demonstrated that after 24h treatment with 2.3 µM of paclitaxel, MSCs were able to package only 0.02–0.04% of the total paclitaxel dose given (corresponding to 0.5–1 nM) into exosomes. This is 100-fold lower than the lowest concentration that we tested in the macrophage polarization assay [38]. 

With respect to specific pathways, the most potent small molecule modulators increased exosome secretion in MSCs by enhancing the expression of genes related to the ESCRT-independent pathway, such as nSMase2 [39,40,41]. nSMase2 is a key regulator of MVBs budding from early endosomes by generating ceramide. Ceramide is essential for exosome formation [29]. Additionally, the small molecule modulators upregulated other genes, such as MITF, Rab27a, and Rab27b, which are known to be essential for the transportation and secretion of exosomes [32,33]. The pathways modulated by the eight upregulated proteins support observations from several groups, as exosomes have been heavily implicated in altering and binding to ECM proteins to promote cell migration and cell adhesion [42,43,44]. The assembly of collagen fibrils, as well as integrin dependent and independent binding, is necessary for the propagation of wound healing responses after injury. Bioinformatic pathway analyses also yielded gene hit counts against laminin-dependent pathways, further supporting our in vitro data, as laminins play a critical role in reepithelization and angiogenesis [45]. The up-regulated proteins used for pathway prediction analysis are directly in line with the angiogenesis, macrophage, and tube formation assay, as they are all involved in exosomal binding to ECM proteins and promotion of wound-healing effects [46,47,48]. Despite these proteins only composing 0.6% of the total protein sample, they are highly enriched (significantly above average protein abundance) and could be of therapeutic benefit in tissue regeneration and wound healing. 

## 5. Conclusions

We have shown that exosome secretion in MSCs can be enhanced by treatment with small molecule modulators. These small molecule modulators did not affect the intrinsic regenerative effects of MSC exosomes and did not significantly alter total exosomal protein expression levels. The proteomic alterations that were observed appear to potentially enhance the MSC exosomes’ therapeutic action. Thus, these compounds could be used to enhance exosome secretion from MSCs for practical application. Further investigation into the structural optimization of small molecule modulators capable of enhancing exosome production is warranted.

## Supplementary Materials

The following are available online at https://www.mdpi.com/2073-4409/9/3/660/s1, Figure S1: Target prediction of the compounds; Figure S2: Assessment of protein abundance change with respect to average abundance value; Figure S3: Polarization of macrophages with compound treatment; Figure S4: Tube formation assay after compound treatment; Table S1: Primer sequences for RT-PCR.

## Abbreviations

MSC	mesenchymal stem cell
FT	fenoterol
NE	norepinephrine
FK	foskolin
MeDA	N-methyldopamine
Mepn	mephenesin
HCF	human cardiac fibroblast
HUVEC	human umbilical vein endothelial cell
BMDM	bone-marrow derived macrophage
MVB	multi-vesicular body
ESCRT	endosomal sorting complex required for transport
