# Supplementary material for: Boosting the Biogenesis and Secretion of Mesenchymal Stem Cell-Derived Exosomes
Source: Cells. 2020 Mar 9;9(3):660. doi: 10.3390/cells9030660 (PMC7140620; doi:10.3390/cells9030660)
Supplement: Supplementary file 1 [file cells-09-00660-s001.pdf]

Boosting the biogenesis and secretion of mesenchymal stem cell-derived exosomes

Jinli Wang [1], Emily E. Bonacquisti[2], Adam D. Brown[2] and Juliane Nguyen[2]*

[1] Department of Biomedical Engineering, University at Buffalo, The State University of New York, Buffalo, NY 14214, USA

[2] Division of Pharmacoengineering and Molecular Pharmaceutics, Eshelman School of Pharmacy, University of North Carolina at Chapel Hill, Chapel Hill, NC 27599, USA.

Supplementary Material

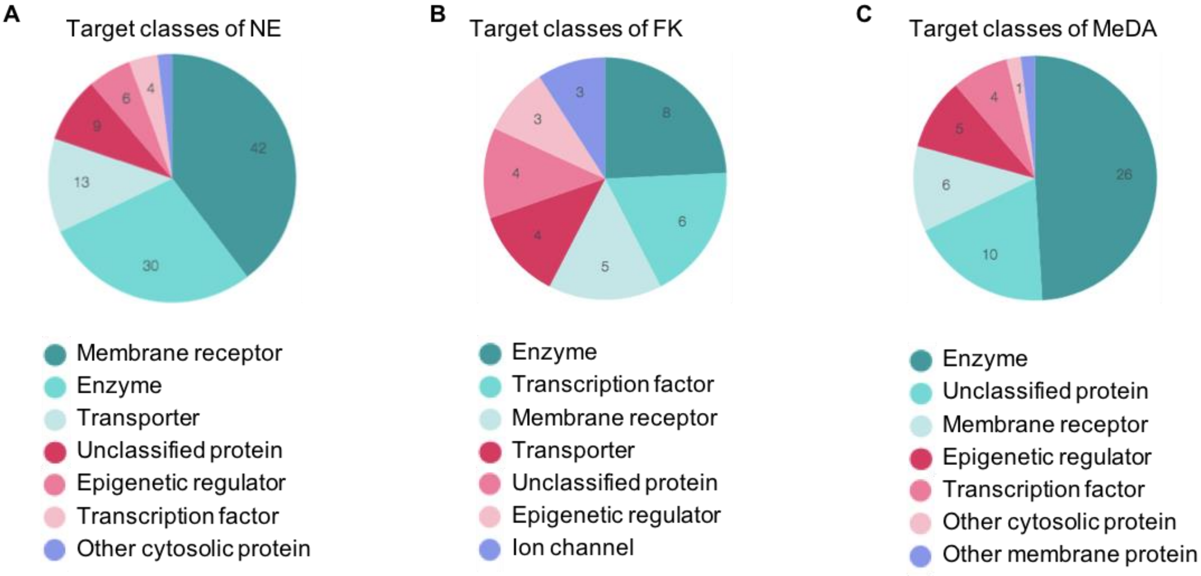

Figure S1. Target prediction of the compounds. CheMBL database and SwissTargetPrediction were used to predict the potential targets of the tested compounds, (**A**) 42 targets of NE are membrane receptors and NE mainly targets different kinds of adrenergic receptors**.** (**B**) FK targets adenylate cyclase, glucagon-like peptide receptor, and glucose transporter, which are related to energy (ATP and glucose) production in cells. (**C**) MeDA targets dopamine receptors and inflammation related proteins.

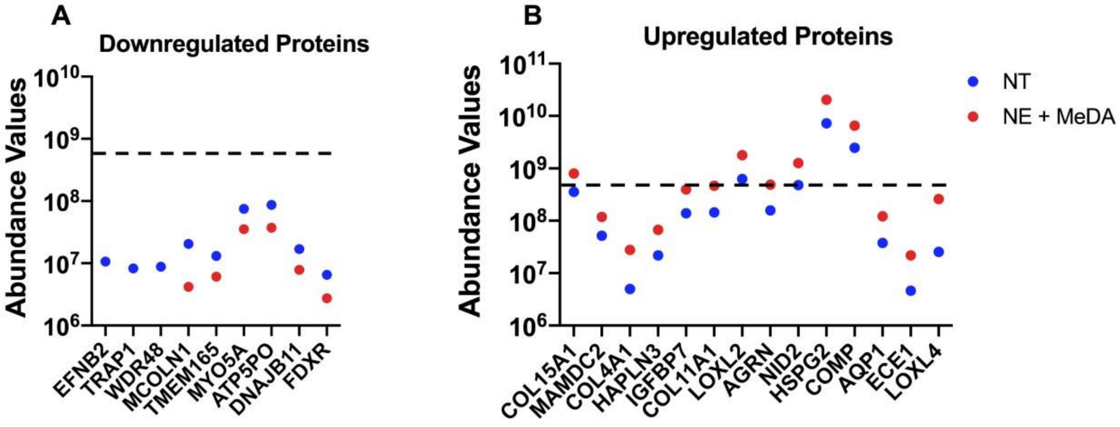

Figure 2. Assessment of protein abundance change with respect to average abundance value. Proteins that were found to be up- (A) or down- (B) regulated between exosomes derived from non-treated MSCs (NT) and MSCs treated with NE and MeDA were plotted with respect to the average protein

abundance (dotted line). Downregulated proteins were found to be in relatively low abundance with respect to the average. Proteins that were upregulated over the average abundance (COL15A1, COL11A1, LOXL2, AGRN, NID2, HSPG2, COMP) were considered for pathway analysis.

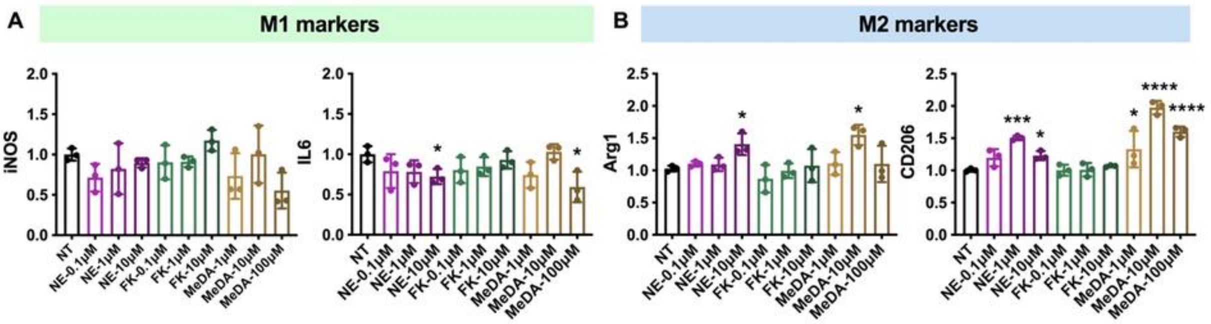

Figure S3. Polarization of macrophages with compound treatment. (**A**) Inflammatory macrophage (M1) markers *iNOS* and *IL6*. **(B)** Anti-inflammatory macrophage (M2) markers *Arg1* and *CD206*. One-way ANOVA with Dunnett's multiple comparison test, * $p < 0.05$, ** $p < 0.01$, *** $p < 0.001$, **** $p < 0.0001$; $n = 3$.

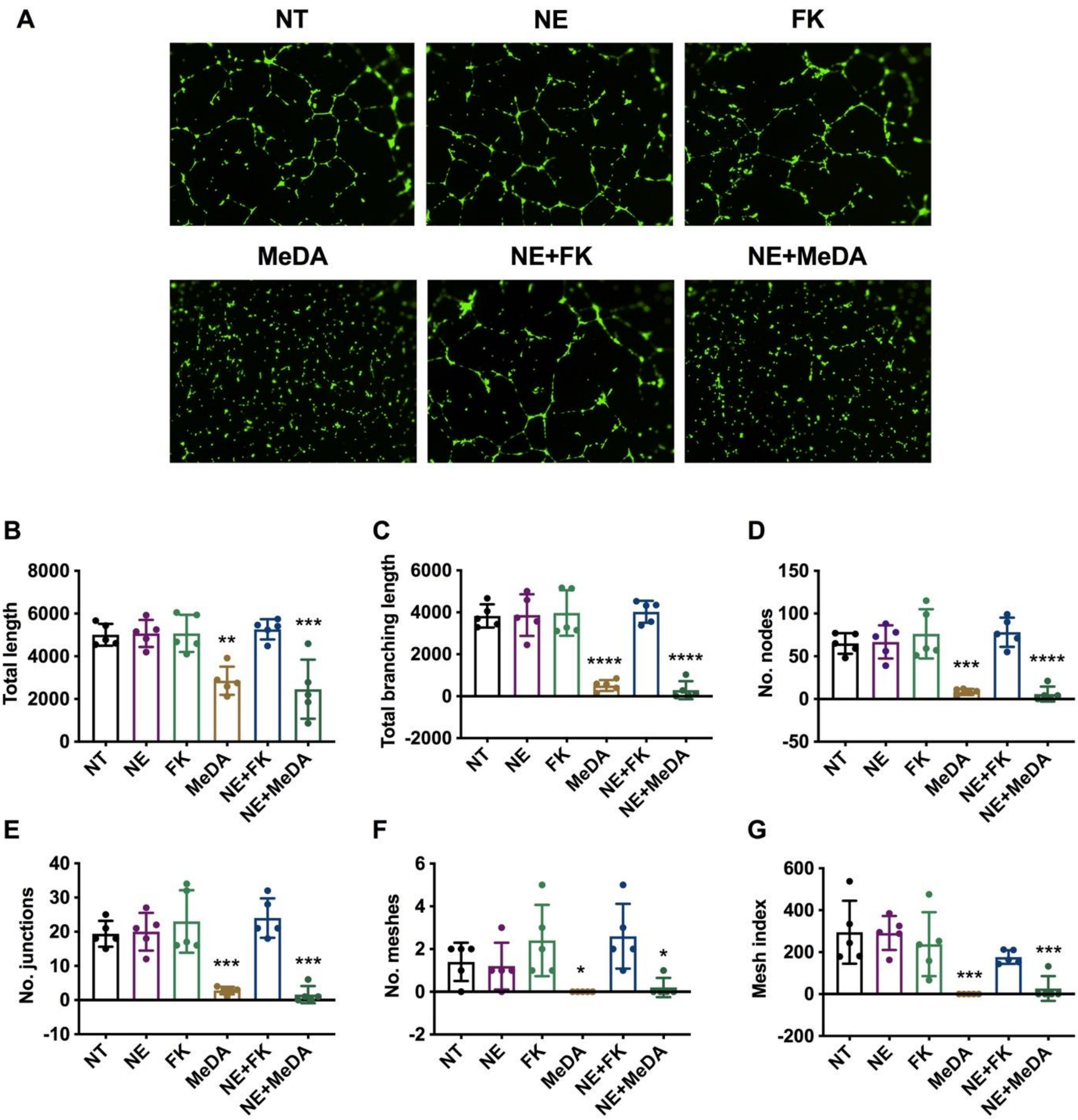

Figure S4. Tube formation assay after compound treatment. **(A)** Representative images of the tube formation changes after compound treatment. **(B–G)** Quantification of the extent of angiogenesis using ImageJ. **(B)** Total tube length quantification**. (C)** Total branching length quantification. **(D)** Respective number of nodes for each condition. **(E)** Number of junctions for each condition. **(F)** Number of meshes. **(G)** Mesh index. One-way ANOVA with Dunnett's multiple comparison test, * $p < 0.05$, ** $p < 0.01$, *** $p < 0.001$, **** $p < 0.0001$; $n = 5$.

Table S1: Primer sequences for RT-PCR.

No.	Gene	5'-3' forward primer	5'-3' reverse primer
1	nSMase2	CAACAAGTGTAACGACGATGCC	CGATTCTTTGGTCCTGAGGTGT
2	Hrs	CTCCTGTTGGAGACAGATTG	CAGGTACAGGATCTTGTTAC
3	Tsg101	GAGAGCCAGCTCAAGAAAATGG	GGGATTGTTCCAGTGAGGTTC
4	Stam1	GATGAATACTGCTGAGGACT	CTGAGAGCCAATAGCTGGGA
5	Alix	CTGGAAGGATGCTTTCGATAAAGG	AGGCTGCACAATTGAACAACAC
6	MITF	AAGGGCTTGCAGAACACCTTA	GCTGGTTTGGACATGGCAAG
7	Rab27a	AGAGGAGGAAGCCATAGCAC	CATGACCATTTGATCGCACCAC
8	Rab27b	GGAACTGGCTGACAAATATGG	CAGTATCAGGGATTTGTGTCTT
9	GAPDH	CAAGGTCATCCATGACAACTTTG	GTCCACCACCCTGTTGCTGTAG
